# Mindfulness and trial-based cognitive therapy for the psychological well-being in the judiciary: A controlled and randomized study protocol

**DOI:** 10.1016/j.mex.2024.103021

**Published:** 2024-10-28

**Authors:** Sara Bitencourt, Bo-Huei Huang, Irismar Reis de Oliveira, Marcelo Demarzo

**Affiliations:** a Federal University of São Paulo (UNIFESP), “Mente Aberta” – Brazilian Center for Mindfulness and Health Promotion, Department of Preventive Medicine, São Paulo, Brazil; b School of Public Health, Faculty of Health, University of Technology, Sydney, Australia; c Federal University of Bahia (UFBA), Department of Neurosciences and Mental Health, Salvador, Brazil

**Keywords:** Mindfulness, Trial-based cognitive therapy, Psychological well-being, Judiciary, Mindfulness-Integrated Trial-Based Cognitive Therapy

## Abstract

**Introduction:**

The judiciary sector faces high absenteeism due to mental health issues, yet applying effective therapies like mindfulness and trial-based cognitive therapy (TBCT) remains unexplored.

**Objectives:**

Develop and apply Mindfulness-Integrated Trial-Based Cognitive Therapy (M-TBCT) in the judiciary to assess its acceptability, feasibility, and efficacy in enhancing psychological well-being and reducing depression, anxiety, and improving self-care.

**Methods:**

Conduct a controlled, randomized study in two phases: Phase 1 involves a pilot study to structure the M-TBCT protocol and test its acceptability and feasibility in the judiciary (*N* = 30); Phase 2, an efficacy trial with 90 participants, comparing M-TBCT to a waitlist. M-TBCT consists of 8 online, individual, weekly sessions. Participants recruited via SRQ-20, with WHO-5, PHQ-9, GAD-7, CD-QUEST and ASAS-R as outcomes.

**Expected outcomes:**

M-TBCT is expected to be acceptable, feasible, and effective in improving psychological well-being, reducing anxiety, depression, and enhancing self-care, with sustained benefits at a 6-month follow-up.

**Conclusions:**

This study introduces a novel psychotherapeutic approach to address mental well-being and self-care in the judiciary.

## Graphical abstract

**Figure:**
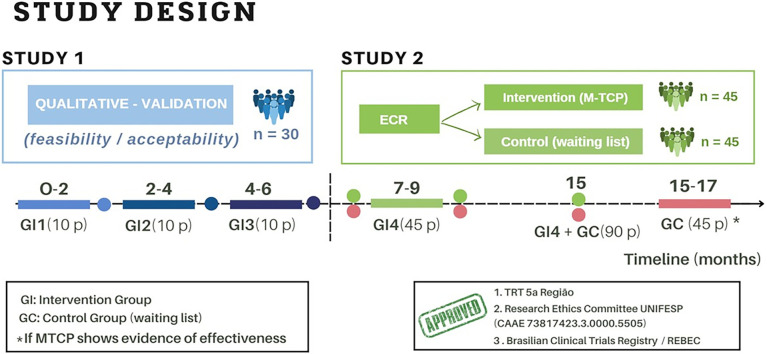

Specifications table

**Table:** 

Subject area:	Psychology
More specific subject area:	Mindfulness and trial-based cognitive therapy
Name of your protocol:	Mindfulness-Integrated Trial-Based Cognitive Therapy
Reagents/tools:	N.A.
Experimental design:	This is a mixed methods study (quantitative-qualitative) divided into two phases: a pilot study to structure the M-TBCT protocol and test its acceptability and feasibility (phase 1) and efficacy trial of M-TBCT, single-blind, with two parallel arms (phase 2).
Trial registration:	Ensaiosclinicos.gov.br: U1111–1304–1781.
Ethics:	This study was approved by the Research Ethics Committee of the Federal University of São Paulo (CAAE 73,817,423.3.0000.5505). Participants will sign the Informed Consent Form online and data confidentiality will be guaranteed.
Value of the Protocol:	1) First randomized and controlled feasibility study that structures a protocol integrating psychotherapeutic tools validated in the literature and three assessment points (baseline, post-intervention and 6-month follow-up) 2) This randomized, controlled study aims to fill a gap in scientific evidence-based interventions to promote well-being in the judiciary 3) Improvements in well-being and self-care are expected, as well as a reduction in levels of anxiety, depression and cognitive distortions.

## Background

Recent psychological research has increasingly emphasized the importance of promoting well-being and positive mental health, aligning with the World Health Organization's definition of health as “a state of complete physical, mental, and social well-being” [1]. Carol Ryff's multidimensional model of psychological well-being, encompassing aspects such as self-acceptance, autonomy, environmental mastery, purpose in life, positive relations with others, and personal growth [2], serves as a foundation for our study.

Mindfulness and cognitive therapy have demonstrated positive effects on mental health in various workplace settings [3,4]. Jon Kabat-Zinn's mindfulness approach, centered on present-moment awareness and non-judgment [5], has been adapted for diverse contexts, including the Mindfulness-Based Health Promotion Protocol (MBHP; [6]). This protocol has shown efficacy in improving mental health across multiple randomized controlled trials [[7], [8], [9]], addressing issues such as chronic pain [10], burnout [11], and obesity [12].

Trial-Based Cognitive Therapy (TBCT), an innovative extension of Cognitive-Behavioral Therapy (CBT) developed by de Oliveira [13], focuses on challenging dysfunctional thoughts, emotions, and behaviors. TBCT's unique courtroom metaphor approach has proven effective in treating a range of mental health conditions, including depression, suicidal ideation [14,15], social anxiety [16], PTSD [17], and OCD [18]. This approach combines traditional CBT techniques with elements from other psychotherapies [19,20].

The judiciary sector, vital for societal function, has witnessed a concerning rise in mental health problems, intensified by the Covid-19 pandemic [21]. Symptoms such as fatigue, mood changes, and sleep disturbances have been reported among judicial staff [22].

In response to these challenges, our study aims to develop and implement Mindfulness-Integrated Trial-Based Cognitive Therapy (M-TBCT) specifically tailored for the judiciary context. Through a pilot phase and a subsequent randomized controlled trial, we will evaluate the acceptability, feasibility, and efficacy of M-TBCT in enhancing psychological well-being, reducing depression and anxiety symptoms, and promoting self-care practices among judiciary professionals.

This research seeks to contribute to the development of targeted interventions that foster a healthier and more resilient judiciary workforce, potentially informing future occupational health initiatives. The primary objectives are to refine the M-TBCT protocol, assess its acceptability and feasibility within the judiciary, and compare its efficacy against a waitlist control group, measuring its impact on key mental health outcomes such as depression, anxiety, cognitive distortions and self-care.

## Description of protocol

### Study design

This study consists of two phases. The first is a pilot study to develop the M-TBCT protocol, assessing its suitability and practicality within the judicial context. The second phase is an efficacy trial using a single-blind design with two groups: an M-TBCT intervention group and a waitlist control group. Inspired by Soler et al. [23], the first phase will involve a sample of 30 participants, divided into three blocks of 10, each receiving M-TBCT in eight weekly one-hour sessions. Participant feedback via a self-report questionnaire will guide protocol refinement.

The second phase, an efficacy trial, will have 90 participants randomly assigned to either the intervention or waitlist group, each receiving the same M-TBCT regimen. The M-TBCT protocol will be administered individually on a weekly basis, with each session lasting 1 h and totaling 8 online sessions.

### Evaluation measures

**Fig. 1:**
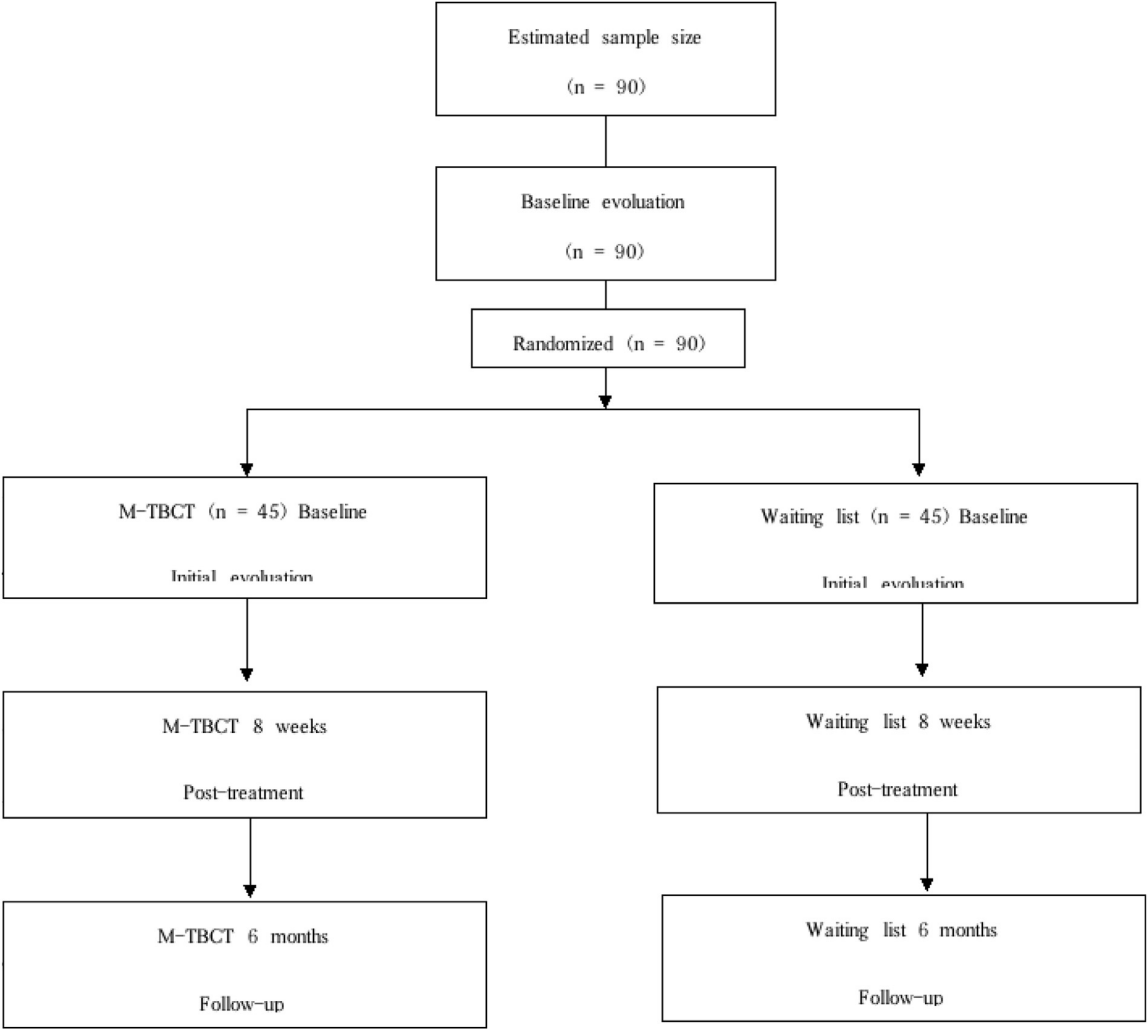
Study flowchart (CONSORT). M-TBCT: Mindfulness-integrated trial-based cognitive therapy.

Assessments will occur pre-intervention (T0), post-intervention (T1), and at a six-month follow-up. These will include questionnaires measuring well-being (WHO-5), depression (PHQ-9), anxiety (GAD-7), reduction in cognitive distortions (CD-QUEST) and self-care (ASAS-R). Post-study, M-TBCT will be offered to the waitlist group if results are favorable. The study stages will follow the CONSORT flowchart [24] depicted in Fig. 1. The study was approved by the Research Ethics Committee of the Federal University of São Paulo (CAAE 73817423.3.0000.5505) and registered in the Brazilian Clinical Trials Registry (REBEC) 〈https://ensaiosclinicos.gov.br/〉.

### Hypotheses

Based on prior research, we hypothesize:

Hypothesis 1 (H1) M-TBCT will demonstrate acceptance and feasibility among judges and public servants in the judiciary.

Hypothesis 2 (H2) M-TBCT will significantly improve psychological well-being, self-care, and reduce anxiety, depression and cognitive distortions compared to the control group.

Hypothesis 3 (H3) The benefits of M-TBCT will be maintained at the six-month follow-up.

### Participants

After the study protocol is publicized through institutional media such as videos, website, and emails, interested individuals will be selected using the SRQ-20. Those scoring 7 points or higher and meeting other eligibility criteria will be invited via email by the researcher as part of a care initiative aimed at learning and practicing evidence-based skills to develop autonomy and self-efficacy in promoting psychological well-being. During this invitation, participants will receive explanations about how the study will work, and those who agree to participate will sign the informed consent form. After completing the self-assessment questionnaires, the first thirty participants will proceed to phase 1 of the study. Upon completion of phase 1, other ninety participants scoring 7 or higher on the SRQ-20 and meeting other eligibility criteria will proceed to phase 2 of the study, being randomized into one of the two arms (M-TBCT or waitlist for treatment).

### Inclusion criteria

Individuals eligible to participate in this study (phases 1 and 2) are adults of both sexes, employees, or judges of TRT5, aged 18 years or older, with a score of 7 or more on the SRQ-20. Participants should be able to read, write, and follow instructions and must have internet access.

### Exclusion criteria

Candidates meeting any of the following criteria will be excluded from participating in this study: self-declaration of severe symptoms of any mental disorder or ongoing organic disease; having a personality disorder, a diagnosis of schizophrenia, psychotic disorder, suicidal ideation, or drug use causing cognitive impairments in attention and concentration; being in the early stages or adjusting to pharmacological and/or psychotherapeutic treatment; and being a regular practitioner of mindfulness, meditation, or similar practices.

### Study settings

Eligible participants may be located in Salvador or any other city within the state covered by TRT5. TRT5, located in Salvador, will be the study headquarters and the headquarters of one of the lead researchers. The Federal University of São Paulo, the academic institution responsible for the study, is also the headquarters of one of the lead researchers and the Federal University of Bahia, the academic institution of another lead researcher.

### Intervention

The presented M-TBCT protocol will be applied individually and online through the Google Meet platform. Sessions will be held weekly, each lasting 1 h, totaling eight sessions over 2 months.

As mentioned, M-TBCT is an integration of two protocols, Mindfulness-Based Health Promotion (MBHP) and Trial-Based Cognitive Therapy (TBCT), which have shown promising results across various populations:

**Mindfulness-Based Health Promotion Protocol (MBHP):** Developed by Dr. Marcelo Demarzo and inspired by Jon Kabat-Zinn's Mindfulness-Based Stress Reduction (MBSR), MBHP embodies the core principles of mindfulness, focusing on present-moment awareness with acceptance and non-judgment [6].

The MBHP aims to cultivate and enhance the state of consciousness regarding internal and external phenomena, thus fostering awareness. The program contributes to improved decision-making, conscious choices, defusion, enhanced socioemotional skills, and well-being. It is designed for application in both clinical (ill) and non-clinical (healthy) populations [6].

The MBHP is a structured program with a limited number of sessions that targets individuals aged 18 years and above. It is recommended for those without severe symptoms, not diagnosed with mental or organic disorders, not undergoing treatment adjustments, and those without personality disorders. The program can be implemented in group or individual settings. The latter format, as explored in this study, involves approximately 8 weekly sessions, each lasting 1 h [6].

This approach is significant because of its adaptability to different cultural contexts and has demonstrated efficacy in diverse populations. For instance, MBHP has been effective in reducing burnout in police officers [11], enhancing the quality of life in older adults [25], and serving as a complementary intervention in obesity treatment [12]. These findings suggest that the versatile nature of MBHP could be beneficial in addressing the unique stressors faced by judiciary professionals.

**Trial-Based Cognitive Therapy (TBCT):** TBCT, developed by Brazilian psychiatrist Irismar Reis de Oliveira, is grounded in cognitive-behavioral therapy and uses experiential techniques to modify dysfunctional thoughts, emotions and behaviors.

The main TBCT techniques employ an analogy to the judicial process, guiding the patient toward decentering through metacognition and enabling a new perspective on negative core beliefs. The TBCT model operates in contrast to the automatic and uncritical mode of functioning that individuals often adopt when negative core beliefs are activated, justifying, in the latter case, the production and maintenance of symptoms. Previous studies have demonstrated the efficacy of TBCT in reducing psychological symptoms compared to traditional CBT [16].

It has been notably effective in treating various psychological disorders such as major depression [15], social anxiety [16,26], PTSD [17], suicidal ideation in major depressive disorder [14], obsessive-compulsive disorder and other adverse conditions [26,27]. This broad range of applicability underscores TBCT's potential effectiveness in the judiciary context, where mental health issues like stress, anxiety, and depression are prevalent.

The M-TBCT protocol (Table 1) integrates mindfulness, compassion, and TBCT strategies to develop metacognitive and socio-emotional skills that enhance well-being, self-care, and health-related quality of life. In simpler and accessible language, participants will be informed that the intervention focuses on learning and practicing evidence-based skills to promote psychological well-being. Mindfulness and self-compassion strategies are derived from the Mindfulness-Based Health Promotion (MBHP) protocol.

**Table 1:** Structure of the mindfulness protocol integrated with trial-based cognitive therapy (M-TBCT).

Sessão*	Interventions/Techniques	Homework assignments
**1. Getting off autopilot**	Cognitive conceptualization diagram (level I) CD-Quest-9 Raisin practice	CD-Quest-9 Text “What *mindfulness* is and isn't” Informal: *Mindfulness* (a part of the meal).
**2. Mindfulness and cognitive distortions**	Practice “Body Scan” Complete Intrapersonal Thought Record (with or without action plan)	Action plan (if built in the session) Text “Why practice *mindfulness*?” Formal: Body Scan
**3. The body and its relationship with thoughts, emotions and behaviors**	Interpersonal Thought Record (RP-Inter) (with or without an action plan) or Participation Grid (for guilt/shame) Practice “Mindful breathing”	Action plan (if created in the session) Text “What to do with the body?” Formal: Mindfulness of breathing.
**4. Facing my fears**	Practice “3 steps” Cognitive conceptualization diagram (level II) Color-coded Symptoms Hierarchy (CCSH)	Action plan created in the session (CCSH) Informal: “3 steps” practice
**5. Expanding mindfulness**	Practice of basic “Sounds and Thoughts” Consensual role-play (CRP) for feared or desired + action plan	Action plan practiced in the session (RPC) Formal: “Sounds and Thoughts” Practice (basic)
**6. Changing Core Beliefs**	Advanced “Sounds and Thoughts” Practice (with tagging and metaphors) TBCT conceptualization diagram (level III) Trial I	Daily positive belief worked on in the session. Formal: “Sounds and Thoughts” Practice (advanced) Informal: bring full attention to walking or other daily physical activity.
**7. Developing positive beliefs**	Trial I (complete or new) Practice “Compassion” (for yourself and for others)	Diary positive belief worked on in the session. Text “Mindfulness and compassion”. Formal: Practice of “Compassion” Informal: choose
**8. Being my own therapist**	Trial II + Compassionate Letter Practice “Self-Compassion” Final feedback	Text “Practice mindfulness without practicing mindfulness. Acceptance of reality.” Curriculum based on the tools learned.

⁎ All sessions will have take-home proposals.

The structure of the M-TBCT protocol envisions a progressive cadence of strategies designed to stimulate autonomy, involving the self-application of learned tools. The intention is to start with the simplest and gradually develop a more gentle and open stance that supports individuals in restructuring dysfunctional cognitions through specific TBCT techniques. The three pillars of the intervention – mindfulness, self-compassion, and TBCT – will operate in an integrated manner from the beginning of the protocol application.

Another highlighted aspect is the introduction of brief and concise homework tasks, favoring the continued exercise of learned tools without generating overload that could discourage the therapeutic process. An alternative strategy is to encourage the construction of a personalized toolkit with the patient, allowing them to have at their disposal the practices that have proven most useful and aligned with their goals, choosing those most suitable for their daily life. Another innovation in this protocol is the possibility for individuals to record, at the end of each session, the practices perceived as most useful and easy to apply in their daily lives.

### Therapist

Both studies anticipate the participation of the psychologist and researcher in this study. She is currently the only professional with completed training in both mindfulness strategies through the Mindfulness-Based Health Promotion (MBHP) protocol from the Open Mind Center at the Federal University of São Paulo and in Trial-Based Cognitive Therapy, as developed by de Oliveira [13].

### Results

In Phase 1, encompassing a pilot study, the acceptability of M-TBCT will assess participants’ perceptions regarding satisfaction with the M-TBCT protocol through a self-report questionnaire. The feasibility of M-TBCT will be verified by recruitment rates (>80 %), session attendance (>60 %), and sample retention at the end of the study (>60 %).

In Phase 2, involving the efficacy of M-TBCT, the primary outcome will be assessed by the increase in well-being, measured through WHO-5. Secondary outcomes include a reduction in depression symptoms (PHQ-9), anxiety (GAD-7), cognitive distortions (CD-QUEST) and an increase in self-care capacity (ASAS-R). All scales will be administered in both groups (M-TBCT and waitlist) before the intervention (baseline assessment), at the end of the 8 sessions (final assessment), and at the 6-month follow-up. Table 2 illustrates the session and assessment flow throughout the study period.

**Table 2:** Session and assessment flow throughout the study period.

Study period				
	Enrollment	Allocation	Post-intervention	Follow-up
**Point in time**	Screening	Baseline	8 weeks	6 months
**Recruitment**				
SRQ-20	X			
Eligibility	X			
Informed consent		X		
Allocation		X		
**Intervention (M-TBCT)**				
**Control group (waiting list)**				
**Assessments**				
**Phase 1**				
Self-report questionnaire			X	
**Phase 2**				
WHO-5		X	X	X
PHQ-9		X	X	X
GAD-7		X	X	X
CD-QUEST		X	X	X
ASAS-R		X	X	X

### Instruments

• Recruitment (Phases 1 and 2)

*Self-Report Questionnaire (SRQ-20):* This self-administered questionnaire, originally developed by the World Health Organization and validated for use in Brazil [28], serves as a screening tool for common mental disorders in Brazilian studies, particularly among worker groups [29]. Comprising 20 "yes" or "no" questions related to symptoms of common mental disorders, a cutoff point of 7 indicates a classification of suspected cases. The validated version for use in Brazil will be employed.

It will also involve the utilization of a self-report questionnaire, enabling individuals to disclose the following non-inclusion criteria: (1) the presence of severe symptoms indicative of an ongoing mental disorder or organic disease; (2) the presence of a personality disorder, a diagnosis of schizophrenia, psychotic disorder, suicidal ideation, or the use of substances causing cognitive impairment affecting attention and concentration; (3) being in the initial phase or undergoing adaptation to pharmacological and/or psychotherapeutic interventions; (4) maintaining regular engagement in mindfulness, meditation, or analogous practices.

• Acceptability and feasibility (phase 1)

Acceptability assesses participants’ perceptions of satisfaction with the M-TBCT protocol. All participants who complete the eight sessions of the program will be invited to complete a self-report questionnaire asynchronously, individually, and online within one week after the intervention. The questionnaire consists of two parts. The first part contains 6 items scored from 0 to 4, where 0 = completely dissatisfied and 4 = completely satisfied, covering the following topics: (1) motivation to participate in the program; (2) therapist's competence; (3) online modality of the intervention; (4) session duration; (5) weekly interval between sessions; (6) quantity and quality of services offered (intervention and support materials); (7) utility of the program in resolving and/or dealing with problems; (8) if the program met expectations; (9) if they would recommend the intervention to others; (10) if they would use the program again; (11) if the program contributed to adhering to what was learned, and (12) overall satisfaction with the program. This part of the questionnaire was adapted from Loucks et al. [30] and Martínez-Sanchis et al. [31]. According to these authors, an intervention is considered well-received when most assessed aspects are recognized by participants as appropriate.

The second part of the questionnaire was adapted from Soler et al. [23], including structured questions about the acceptability and feasibility of the M-TBCT protocol, aiming to cover: (1) the respondent's understanding of well-being and self-care; (2) verifying if and how the protocol contributed to the promotion of well-being and self-care in their daily lives; (3) identifying the practices learned in the protocol that most contributed to the promotion of well-being and self-care in daily life; (4) verifying if there were any challenges that the participant encountered in adhering to the protocol sessions and including practices in daily life; and (5) identifying if there were any practices learned in the protocol that were easier to include in daily life.

According to the literature, the feasibility of an intervention requires meeting at least three of the following indicators: (1) recruitment rate greater than 80 % of the sample size; (2) retention rate equal to or greater than 60 % measured by participation in the final follow-up assessment; (3) attendance rate equal to or greater than 60 % of the total sessions; and (4) intervention evaluation [32].

Previous studies have found good acceptability and feasibility of mindfulness-based interventions, for example, for the self-regulation of blood pressure in hypertensive patients [30]. However, until now, no studies have been identified that verify the acceptability and feasibility of a mindfulness and a TBCT protocol for the main outcome proposed to be measured in this study (psychological well-being), neither with reference to the judiciary.

• Primary Outcome (Phase 2)

The World Health Organization Five (WHO-5) is a concise, self-administered scale developed by the WHO to evaluate subjective well-being. It is widely used as an outcome measure in clinical trials and has undergone various validity studies for use in Brazil. When applied in clinical trials, the WHO-5 effectively captures improvements in well-being resulting from both pharmacological and non-pharmacological interventions across diverse conditions and contexts [33]. The scale comprises five questions organized in a Likert-type scale ranging from 5 (all the time) to 0 (never). Raw scores range from 0 (absence of well-being) to 25 (maximum well-being). It is recommended to multiply the raw score by 4, yielding a scale from 0 (absence of well-being) to 100 (maximum well-being). According to the WHO guidelines, a cutoff score of 50 indicates screening for clinical depression [33].

• Secondary Outcomes (Phase 2)

The Patient Health Questionnaire-9 (PHQ-9) serves as a self-report tool for the brief assessment, diagnosis, and monitoring of depression, aligning with the DSM-IV criteria and reflecting experiences over the past two weeks. Comprising nine items, the questionnaire uses a Likert-type scale with four points ranging from 0 (not at all) to 3 (nearly every day) . A cutoff score of 10 indicates the presence of signs and symptoms of major depression [34]. We will use the validated version tailored for use in Brazil.

The Generalized Anxiety Disorder-7 (GAD-7) serves as a self-report instrument for the concise assessment, diagnosis, and monitoring of anxiety, aligning with the DSM-IV criteria and encompassing the preceding two weeks. Developed by Spitzer et al. [35], consisting of seven items, the questionnaire employs a Likert-type scale with four points ranging from 0 (not at all) to 3 (nearly every day). A cutoff score of 10 indicates the presence of signs and symptoms of generalized anxiety disorder [34]. We will use the validated version designed for Brazil.

The Cognitive Distortions Questionnaire – 9 items (CD-QUEST-9) this is a brief self-report 9-item version [36] of the original 30-item tool developed and validated by de Oliveira et al. [37]. The short version consists of nine cognitive distortions, evaluated in terms of intensity and frequency of occurrence in the last week when completing the questionnaire. The frequency-intensity cross-classification for each of the 9 cognitive distortions includes frequency options such as “No (It did not occur),” “Occasional (1–2 days during the past week),” “Much of the time (3–5 days during the past week),” and “Almost all the time (6–7 days during the past week).” Intensity options include “A little (Up to 30 %),” “Much (31–70 %),” and “Very much (>70 %).”

The Revised Self-Care Assessment Scale (ASAS-R) [38] assesses the levels of health-related self-care capacity within the Brazilian population. It focuses on health-promoting behaviors developed over the course of one's life, not solely when health issues arise. The scale consists of 15 items evaluated using a Likert-type scale ranging from 1 (strongly disagree) to 5 (strongly agree). It demonstrates a good fit and reliability with the three-factor model of the original scale, namely: Factor 1: Having the capacity for self-care (items 1, 2, 3, 5, 6, and 10); Factor 2: Developing the capacity for self-care (items 7, 8, 9, 12, and 13); and Factor 3: Lack of capacity for self-care (items 4, 11, 14, and 15). Damásio and Koller [38] validated its use in Brazil.

The outcome measures will be administered in an online, individual, and asynchronous format, applied before the intervention (T0), after the intervention (T1), and at the 6-month follow-up.

### Data handling

All study-related information will be stored in secure folders with limited access. Paper-based data collection forms will contain only participant numbers to maintain confidentiality and will be stored in a locked cabinet in an area with limited access. Electronic data files will be password protected. The list linking participant numbers and personal information will be stored in a separate password-protected file. Access will be restricted to the research team or designated personnel for 5 years after inclusion. The study has low negligibility risks; therefore, no data monitoring committee will be assigned. Only the first author and lead researchers, or persons assigned by them, will have access to the final dataset. The authors of the final trial report will make substantial contributions to the design, conduct, interpretation, and reporting of the trial. The study findings will be disseminated through publications and scientific presentations, preserving participant confidentiality. Participants and the data-collecting institution will receive copies of the study results.

### Sample size (Phases 1 and 2)

The sample will consist of magistrates and public servants from the judiciary who score a minimum of 7 points on the recruitment instrument (SRQ-20), which is available for online completion throughout the regional jurisdiction via an electronic platform integrated into TRT5’s intranet.

The sample size for Phase 1 of the study was determined with Soler et al. [23] as a reference, considering a minimum of 30 participants. For Phase 2, the study primarily referred to a meta-analysis conducted by Khoury et al. [39], which assessed the effectiveness of 8-week mindfulness-based interventions in healthy individuals and found an average dropout rate of 10 %.

To establish statistical significance, a sample size of 72 individuals was estimated using G*Power 3.1.9.2, based on a one-tailed *t*-test for independent samples with α=0.05, β=0.20, and Cohen's *d* = 0.6. Considering an approximate dropout rate of 25 %, which is higher than that found in similar studies, an estimated recruitment of approximately 90 eligible individuals for the intervention and control groups is anticipated.

### Randomization and sequence generation (Phase 2)

The randomization process, via www.random.org (accessed on December 6, 2023), will be conducted using a computer-generated list of random numbers by the psychologist and lead researcher. After meeting the eligibility criteria and confirming interest in participating in the study, the participant will be invited by the psychologist-researcher to start the intervention (M-TBCT) or wait on the waiting list. Throughout the study, the participant will be informed by the psychologist about the asynchronous assessment deadlines (pre and post-intervention and at the 6-month follow-up).

### Implementation (Phase 2)

Based on the random list, after the participant has signed the informed consent form and been assessed for inclusion criteria and initial measures, they will be informed by the psychologist and lead researcher about starting the intervention or waiting on the waiting list. Those on the waiting list will be informed about their right to access the M-TBCT protocol at the end of the study if favorable results are obtained.

### Randomization and blinding (Phase 2)

Participants meeting the eligibility criteria will be randomly allocated (1:1) to M-TBCT or the waiting list. Randomization is based on a random number service (http://www.random.org, accessed on December 6, 2023), and participants meeting eligibility criteria will be informed by the psychologist and lead researcher.

### Statistical methods (Phases 1 and 2)

In Phase 1 of the study, a mixed methods approach will be employed, aligning with current recommendations for good clinical practice in feasibility analysis. These recommendations suggest that quantitative analyses should be primarily descriptive [[32], [40]]. In Phase 1, participant characteristics will be quantitatively described, while satisfaction with the program and feedback on it will be extracted from the self-report instrument and qualitatively grouped into major themes covered by the instrument. NVivo version 12 software will be used to organize and process the data.

Following the described results, an interpretative analysis and discussion of the findings will be conducted, drawing on the adopted literature. Qualitative information extracted from this instrument will also be used to explore the acceptability of the M-TBCT protocol among the target audience. An analysis predicting participant characteristics that increase or decrease adherence to M-TBCT will also be performed. According to Bowen [41], it is valid to emphasize that qualitative research methods are useful tools for understanding institutional culture.

In phase 2, categorical variables will be presented in numbers and percentages, and continuous variables will be expressed as mean values with standard deviation. Data normality will be assessed using Kolmogorov-Smirnov and Shapiro-Wilk tests. Variance homogeneity assumption will be tested with Levene's test. If the data does not meet the normality test, Bootstrapping procedures will be employed (1000 resamplings; 95 % BCa CI) to enhance result reliability, correcting for non-normal sample distribution and group size differences, and provide a 95 % confidence interval for mean differences [42].

Randomization success will be determined by no significant differences between the groups regarding demographic and clinical characteristics. Continuous variables will be analyzed using Student's *t*-test, and categorical variables will be analyzed using chi-square or Fisher's exact tests. Longitudinal data (T0, T1, and T2) for both intervention and passive control (waitlist) groups will be tracked using the repeated measures Student's *t*-test. Spearman's correlation test will assess variable correlations. M-TBCT program efficacy will be evaluated using repeated measures ANOVA to examine the main effects of time and treatment and their interaction. McNemar's test will assess changes in mental disorder risk classification after M-TBCT intervention. Effect sizes (Cohen's d) and 95 % CI will be calculated for intra- and inter-group comparisons (intervention and waitlist). The Bonferroni correction will be applied to adjust for various statistical tests.

The data will be analyzed through intention-to-treat (ITT), including all randomized participants in their initially assigned group, regardless of completing the treatment protocol. Per-protocol analysis will exclude participants with fewer than four sessions (50 % of the M-TBCT program). Attrition analysis (As-treated) will compare those who completed the protocol with those who responded only to the initial questionnaire. This study employs mixed-effects linear models (MLMs) for statistical analysis. Fixed variables, including depression, anxiety, and self-care, will be incorporated, whereas random variables will be introduced to capture intra-individual variation over time. This will enable modeling of unexplained individual differences not accounted for by the fixed variables, providing a robust approach to understanding the relationships between these variables and well-being throughout the study.

Mediation analysis will be employed to examine the potential interaction among variables, notably depression (PHQ-9), anxiety (GAD-7), cognitive distortions (CD-QUEST) and self-care (ASAS-R), and the primary outcome, represented by well-being (WHO-5). These independent variables will be assessed for their direct impact on well-being, while sociodemographic variables such as gender, age, socioeducational level, income, marital status, number of children, occupation, and working hours will be treated as covariates in the mediation analysis to control for potential confounding factors. All statistical analyses will be conducted using SPSS 28.0 software, with a significance level of 5 % for all study hypotheses.

### Ethical considerations

This study is currently under review by the Research Ethics Committee involving Human Subjects at the Federal University of São Paulo (CAAE: 73,817,423.3.0000.5505). Upon approval, the study will be registered with the Brazilian Clinical Trials Registry (REBEC), which is available at https://ensaiosclinicos.gov.br/.

## Protocol validation

### Discussion

To the best of our knowledge, this study represents a novel approach for integrating mindfulness and TBCT into a structured protocol specifically for the judiciary. Addressing the high prevalence of mental disorders in this sector, which is a leading cause of work-related absenteeism, our protocol aims to offer suitable psychotherapeutic solutions to enhance the well-being of judges and public servants.

We anticipate that our study will contribute substantially to scientific knowledge, guiding the selection and implementation of effective mental health interventions within healthcare systems, particularly in occupational settings such as the judiciary. The study's findings are expected to promote a culture of autonomy and self-efficacy, which are crucial for enhancing well-being, self-care, and productivity .

It is worth mentioning that Peuker et al. [43] emphasized the importance of seeking scientific evidence and its articulation in clinical practice. According to her, this articulation enables the appropriate provision of health demands in various contexts, generating positive impacts on the public system. These impacts often result from the implementation of interventions that are viable, well-accepted, and effective for their intended purposes.

The integration of MBHP and TBCT in M-TBCT is a novel approach particularly suited to the judiciary sector, where the nature of work can lead to unique psychological challenges. The judiciary's high-stress environment and the critical nature of the work undertaken by judges and public servants make this sector a critical area for the implementation of effective mental health interventions [[44], [45], [46]].

Furthermore, the online delivery format of M-TBCT is particularly pertinent in the context of the recent global shift toward remote work and digital health care solutions, a trend accelerated by the COVID-19 pandemic. This mode of delivery could overcome barriers to accessing mental health care in the judiciary, such as time constraints and stigma [47].

Given the efficacy of MBHP and TBCT in various settings and the unique demands of the judiciary sector, M-TBCT could offer a valuable tool for improving mental health and well-being among judiciary professionals. The proposed study will contribute to the literature by exploring the acceptability, feasibility, and effectiveness of this integrated approach in a new context, potentially providing a model for similar interventions in other high-stress professional environments.

### Limitations

On the other hand, while pioneering in its approach and methodology, this study is subject to several limitations that should be considered when interpreting its future results [48,49]:

1. **Online Intervention Format**: The intervention will be delivered online, which, while advantageous in terms of accessibility, may can influence participant engagement and the efficacy of the therapy. Differences in digital literacy, internet access quality, and the absence of face-to-face interaction could impact the outcomes.
2. **Sample Specificity**: The study focuses on judges and public servants within the judiciary, which may limit the generalizability of the findings to other professional groups or sectors. The unique stressors and work environment of the judiciary might mean the intervention's effectiveness could vary in different contexts.
3. **Self-Report Measures**: The reliance on self-report measures for assessing psychological well-being and mental health symptoms could introduce biases such as social desirability or self-assessment inaccuracies. Objective measures, if available, could complement these findings.
4. **Control Group Design**: While a controlled and randomized design will be used, the nature of the control group and potential differences in their experiences compared to the intervention group might influence the study's results.
5. **Short-Term Follow-Up**: The follow-up period of this study is relatively short-term. Long-term effects of the intervention, which are crucial for understanding its sustainability and enduring impact, remain unknown.
6. **Potential Confounders**: There might be unmeasured confounding variables that could affect the outcomes, such as personal life stressors, varying levels of work pressure, or pre-existing mental health conditions.

These limitations highlight the need for further future research in this area, including long-term studies with diverse populations and varied intervention formats, to further validate and extend the findings of this study.

## Conclusions

In conclusion, the M-TBCT protocol aims to improve the psychological well-being of judicial professionals, potentially leading to reduced levels of anxiety and depression and enhanced self-care capacity. Should the protocol prove effective, it could pave the way for broader applications in controlled studies, contributing to the body of evidence supporting the efficacy of online mindfulness and cognitive therapy interventions. This pioneering approach in the judiciary could have far-reaching impacts on societal well-being.

It is hoped that the M-TBCT protocol will be viable, accepted, and effective for improving the psychological well-being of judges and public servants in the judiciary, leading to a reduction in anxiety and depression levels and an increase in self-care capacity in the target audience, with maintenance of results in the assessed follow-up (6 months).

If it shows favorable results, the expectation is that the M-TBCT protocol will be applied to larger samples through controlled and randomized studies, compared to other interventions with scientifically established efficacy, such as scientifically established protocols that inspired the construction of M-TBCT: MBHP and TBCT.

The results will also help expand the body of evidence on the efficacy of online mindfulness and TBCT interventions, as these types of therapies are increasingly used but still lack evidence in the judiciary system. Finally, the results may point to a viable and pioneering psychotherapeutic strategy in the judiciary context, which could be expanded to other regions with a probable favorable impact on society, the direct beneficiary of the services provided.

## CRediT author statement

All authors: Conceptualization, Formal analysis, Methodology, Validation, Writing - original draft, Writing - review and editing. De-Oliveira I.R., Demarzo M: In addition to the above functions, also supervision.

## Declaration of Competing Interest

The authors declare that they have no known competing financial interests or personal relationships that could have appeared to influence the work reported in this paper.

## Data Availability

No data was used for the research described in the article.
